# Supplementary material for: A prospective pilot study assessing levels of preoperative physical activity and postoperative neurocognitive disorder among patients undergoing elective coronary artery bypass graft surgery
Source: PLoS One. 2020 Oct 13;15(10):e0240128. doi: 10.1371/journal.pone.0240128 (PMC7553306; doi:10.1371/journal.pone.0240128)
Supplement: S1 Table — (DOCX) [file pone.0240128.s001.docx]

**S1 Table**  Components of the Short Questionnaire to Assess Health (SQUASH)

| **Sections of the SQUASH** | **Inquired activities** |
| --- | --- |
| *Travel to and from work* | Walking to/from work or school |
|  | Cycling to/from work or school |
| *Physical activity at work or school* | Light or moderate intensity activities |
|  | Vigorous intensity activities |
| *Household activities* | Light or moderate intensity activities |
|  | Vigorous intensity activities |
| *Free time activities* | Walking |
|  | Cycling |
|  | Gardening |
|  | DIY activities |
|  | Additional sport(s) (Max. 4 activities) |
| *Total amount of time* | Combination of cycling, DIY, gardening, sport and other physical activities |
