# Supplementary material for: A prospective pilot study assessing levels of preoperative physical activity and postoperative neurocognitive disorder among patients undergoing elective coronary artery bypass graft surgery
Source: PLoS One. 2020 Oct 13;15(10):e0240128. doi: 10.1371/journal.pone.0240128 (PMC7553306; doi:10.1371/journal.pone.0240128)
Supplement: S2 Table — (DOCX) [file pone.0240128.s002.docx]

**S2 Table** Different intensity categories for physical activities based on age and Metabolic Equivalent of Task (MET) according to values assigned in the Ainsworth compendium for physical activity.

|  | **Ainsworth and Dutch norms for the intensity of an activity** | | |
| --- | --- | --- | --- |
| **Age** | **Light intensity** | **Moderate intensity** | **Vigorous intensity** |
| **<18 years** | <5 MET | 5-8 MET | >8 MET |
| **18-55 years** | <4 MET | 4-6.5 MET | >6.5 MET |
| **>55 years** | <3 MET | 3-5 MET | >5 MET |
