# Supplementary material for: A prospective pilot study assessing levels of preoperative physical activity and postoperative neurocognitive disorder among patients undergoing elective coronary artery bypass graft surgery
Source: PLoS One. 2020 Oct 13;15(10):e0240128. doi: 10.1371/journal.pone.0240128 (PMC7553306; doi:10.1371/journal.pone.0240128)
Supplement: S3 Table — (DOCX) [file pone.0240128.s003.docx]

**S3 Table** Univariate logistic regression for patients meeting the preoperative physical activity recommendations according to the combinorm.

|  | ***Univariate analyses for meeting physical activity recommendations (n=100)*** |  |
| --- | --- | --- |
| ***Factor*** | ***OR (95% CI)*** | **p value** |
| Age | 0.964 (0.907-1.024) | 0.237 |
| Gender (male) | 0.824 (0.200-3.387) | 0.788 |
| BMI | 1.045 (0.951-1.149) | 0.358 |
| Logistic Euroscore | 1.017 (0.893-1.158) | 0.802 |
| Pre-existing comorbidities |  |  |
| Diabetes Mellitus (yes) | 1.473 (0.562-3.860) | 0.431 |
| Hypertension (yes) | 0.649 (0.258-1.633) | 0.359 |
| COPD (yes) | 0.677(0.136 – 3.373) | 0.634 |
| Respiratory (yes) | 0.940 (0.233 -3.796) | 0.931 |
| Other (yes) | 0.667 (0.249-1.804) | 0.425 |
| Preoperative HADS anxiety score | 1.467 (0.408-5.273) | 0.557 |
| Preoperative HADS depression score | 2.000(0.441-9.073) | 0.369 |
| Impaired Hand-grip strength (yes) | 0.576 (0.175-1.890) | 0.363 |
| *CI = confidence interval; CPB = caiopulmonary bypass; HADS = Hospital anxiety and depression scale; OR = odds ratio.* | | |
