# Supplementary material for: A prospective pilot study assessing levels of preoperative physical activity and postoperative neurocognitive disorder among patients undergoing elective coronary artery bypass graft surgery
Source: PLoS One. 2020 Oct 13;15(10):e0240128. doi: 10.1371/journal.pone.0240128 (PMC7553306; doi:10.1371/journal.pone.0240128)
Supplement: S4 Table — (DOCX) [file pone.0240128.s004.docx]

**S4 Table** Pre-operative physical activity and frailty assessments

|  | **All patients (n=100)** | **Physically**  **active**  **(n=76)** | **Physically inactive**  **(n=24)** |
| --- | --- | --- | --- |
|  |  |  |  |
| **Handgrip strength** | *n=97* | *n=76* | *n=21* |
| Left as a percentage of age-gender matched controls | 107.3 (22.3) | 108.4(22.5) | 105.8(21.0) |
| Right as a percentage of age-gender matched controls | 103.4 (21.9) | 105.8 (21.0) | 95.0(23.5) |
| Impaired handgrip strength (<85%) | 15 (15%) | 10(13.2%) | 5 (20.8%) |
|  |  |  |  |
| **Get up and go test** | *n=96* | *n=73* | *n=23* |
| > 12 seconds | 5 (5.2%) | 3 (4.1%) | 2 (8.7%) |
| *Data are presented as number (%), unless otherwise indicated. IQR = interquartile range; NNGB = Dutch Norm of Healthy Activity; SD = standard deviation | | | |
