# Supplementary material for: A prospective pilot study assessing levels of preoperative physical activity and postoperative neurocognitive disorder among patients undergoing elective coronary artery bypass graft surgery
Source: PLoS One. 2020 Oct 13;15(10):e0240128. doi: 10.1371/journal.pone.0240128 (PMC7553306; doi:10.1371/journal.pone.0240128)
Supplement: S5 Table — (DOCX) [file pone.0240128.s005.docx]

**S5 Table** Univariate regression analyses for Postoperative Neurocognitive Disorder (PNCD)

|  | **Univariate analyses for PNCD (n=50)** |  |
| --- | --- | --- |
| **Factor** | **OR (95% CI)** | **p value** |
| Physically active (combinorm) | 0.357 (0.40-3.222) | 0.359 |
| Total Activity Score (n=47) | 1.000 (1.000-1000) | 0.382 |
| CPB (yes) | 0.411 (0.113-1.494) | 0.177 |
| Age; years | 1.024 (0.958-1.094) | 0.484 |
| Education (n=25) | 1.327 (0.698-2.524) | 0.388 |
| Preoperative handgrip strength of dominant hand | 1.011 (0.978-1.044) | 0.520 |
| Postoperative HADS – anxiety score | 0.981  (0.807-1.192) | 0.846 |
| Postoperative HADS– depression score | 1.064 (0.836-1.355) | 0.614 |
| Postoperative complications (yes) | 0.735 (0.202-2.674) | 0.641 |
| CI = confidence interval; CPB = cardiopulmonary bypass; HADS = Hospital anxiety and depression scale; OR = odds ratio; PNCD = postoperative neurocognitive disorder. Postoperative complications include but are not limited to repeat surgery, delirium, cerebrovascular accidents and atrial fibrillation. | | |
