# Supplementary material for: A prospective pilot study assessing levels of preoperative physical activity and postoperative neurocognitive disorder among patients undergoing elective coronary artery bypass graft surgery
Source: PLoS One. 2020 Oct 13;15(10):e0240128. doi: 10.1371/journal.pone.0240128 (PMC7553306; doi:10.1371/journal.pone.0240128)
Supplement: S6 Table — (DOCX) [file pone.0240128.s006.docx]

**S6 Table**. Standardized scores for each cognitive domain prior to surgery, physically active vs. physically inactive.

|  | **Physically active** | **Physically inactive** | |  | | **p value** | |
| --- | --- | --- | --- | --- | --- | --- | --- |
| **Standardized Scores** | Baseline  (n=66)  Mean (SD) | Baseline  (n=21)  Mean (SD) |  | | Baseline | |  |
| Psychomotor Speed | 100.36 (10.96) | 100.05(10.86) |  | | 0.910 | |  |
| Selective Attention | 98.50 (8.75) | 97.35 (14.03) |  | | 0.727 | |  |
| Long-term Memory | 93.23 (7.97) | 91.46 (8.26) |  | | 0.384 | |  |
| Working Memory | 100.48 (10.64) | 97.52 (9.51) |  | | 0.309 | |  |
| *A higher score reflects a better performance. A score of 100 is the age-matched population mean, and every 10 points divergence is one SD of the age-matched population. | | | | | | |  |
