# Supplementary material for: A prospective pilot study assessing levels of preoperative physical activity and postoperative neurocognitive disorder among patients undergoing elective coronary artery bypass graft surgery
Source: PLoS One. 2020 Oct 13;15(10):e0240128. doi: 10.1371/journal.pone.0240128 (PMC7553306; doi:10.1371/journal.pone.0240128)
Supplement: S7 Table — (DOCX) [file pone.0240128.s007.docx]

**S7 Table.** Preoperative neuropsychological and wellbeing test results

| **Test** | **Preoperatively active** | **Preoperatively inactive** | **p value** |
| --- | --- | --- | --- |
| **Preoperative screening HADS** | *n=75* | *n=24* |  |
| Anxiety, number (%) | 9 (12.0%) | 4 (16.7%) | 0.556 |
| Depression, number (%) | 5 (6.7%) | 3 (12.5%) | 0.361 |
| **RAND-36** | *n=75* | *n=23* |  |
| Physical function | 70 (58.75-90) | 50 (25-77.5) | **0.004** |
| Social function | 81.25 (62.5 -100) | 62.5 (43.75-81.25) | **0.005** |
| Role limitation -physical | 50 (0-100) | 0(0-25) | **0.002** |
| Role limitation – emotional | 100(33.33-100) | 0(0-66.66) | **<0.001** |
| Mental health | 80(64-88) | 76(64-88) | 0.947 |
| Vitality | 65(45-75) | 55(35-60) | **0.010** |
| Pain | 78.79(67.34-100) | 77.55(34.88-93.877) | 0.090 |
| General health | 60(45-70) | 50(40-62.5) | **0.045** |
| Health changes | 25(25-50) | 25(0-35) | 0.088 |
|  |  |  |  |
| **EQ5D** | *n=76* | *n=24* |  |
| Mobility | 1 (1-2) | 2(1-2) | 0.063 |
| Selfcare | 1 (1-1) | 1 (1-1) | 0.702 |
| Usual activities | 1 (1-2) | 1 (1-2) | 0.133 |
| Pain and discomfort | 2 (1-2) | 2 (1-2) | 0.765 |
| Anxiety and depression | 1 (1-2) | 1 (1-2) | 0.842 |
| Transformed EQ5D score | 0.843 (0.773-1) | 0.8070 (0.6510-0.9465) | 0.211 |
| **WHODAS** | *n=41* | *n=19* |  |
| D1 Understanding and communicating | 4.17 (0-10.415) | 2.085 (0-20.83) | 0.883 |
| D2 Getting around | 5 (0-20) | 10 (5-20) | 0.260 |
| D3 Self care | 0 (0-6.25) | 0 (0-6.25) | 0.751 |
| D4 getting along with people | 5 (0-20) | 5 (0-20) | 0.886 |
| D5 life activities | 0 (0-10.994) | 3.13 (0-18.75) | 0.478 |
| D6 Participation in society | 15.63 (6.25-23.44) | 15.63 (9.38-21.88) | 0.894 |
| Overall | 5.8 (3.87-12.1) | 9.34 (3.13-16.6) | 0.386 |
| *Data are presented as median (interquartile range), unless otherwise indicated. EQ5D = EuroQol-5D; RAND-36 = Research and Development-36; WHODAS = World Health Organisation, Disability Assessment Schedule. | | | |
